# Supplementary material for: mTOR Inhibitors Modulate the Physical Properties of 3D Spheroids Derived from H9c2 Cells
Source: Int J Mol Sci. 2023 Jul 14;24(14):11459. doi: 10.3390/ijms241411459 (PMC10380298; doi:10.3390/ijms241411459)
Supplement: Supplementary file 1 [file ijms-24-11459-s001.zip › ijms-2440953-supplementary.pdf]

Supplemental Table S1 Sequences of primers of qPCR

		Sequence	Exon Location	RefSeq Number
rat RPLP0	Probe	5'-/56-FAM/CCTGTCTTC/ZEN/CCTGGGCATCACG/3IABkFQ/-3'	1-2	NM_022402
	Primer2	5'-TGTCTGCTCCCACAATGAAG-3'		
	Primer1	5'-CAATCCCTGACGCACCG-3'		
rat COL1A1	Probe	5'-/56-FAM/CCGGAGGTC/ZEN/CACAAAGCTGAACA/3IABkFQ/-3'	-	NM_053304
	Primer2	5'-CATTGTGTATGCAGCTGACTTC-3'		
	Primer1	5'-CGCAAAGAGTCTACATGTCTAGG-3'		
rat COL4A1	Probe	5′-/TCACCTTTC/ZEN/TGACCCTTTTCTCCTGTTG/3IABkFQ/-3′	13-15	NM_001135009
	Primer2	5′-GTTCACCTTTCTCTCCATATCCTG-3′		
	Primer1	5′-GGACAGGCACAAGTTAAGGAA-3′		
rat COL6A1	Probe	5′-/56-FAM/CAGGTTGCG/ZEN/GTCACATCGGTAGT/3IABkFQ/-3′	2-3	XM_215375
	Primer2	5'-ATCTCCACCTCGTCACTGTA-3'		
	Primer1	5'-CCTTTACCAAGCGTTTCATCG-3'		
rat FN1	Probe	5′-/56-FAM/CGCCGTGGT/ZEN/CCTAACAAATCTCCTG/3IABkFQ/-3′	26-27	NM_019143
	Primer2	5'-GATGCTCTCATGCTGTTCGTA-3'		
	Primer1	5'-CGCCGTGGT/ZEN/CCTAACAAATCTCCTG-3'		
rat αSMA	Probe	5'-/56-FAM/ACGACATGG/ZEN/AAAAGATCTGGCACCA/3IABkFQ/-3'	3-4	NM_031004
	Primer2	5'-ACGCGAAGCTCGTTATAGAAG-3'		
	Primer1	5'-GACCCTGAAGTATCCGATAGAAC-3'		
rat Cx43	Probe	5′-/56-FAM/AGTGAAAGA/ZEN/GAGGTGCCCAGACATG/3IABkFQ/-3′	1-2	NM_012567
	Primer2	5′-GGTGGAGTAGGCTTGGAC-3′		
	Primer1	5′-CCTTTGACTTCAGCCTCCAA-3′		
rat ZO1	Probe	5′-/56-FAMCATCCGCCT/ZEN/TCCCCTCAGAGAC/3IABkFQ/-3′	17-18	NM_001106266
	Primer2	5′-ACAGGTAGGACAGACGATCA-3′		
	Primer1	5′-TCAGCAGCAACAGAACCAG-3′		
rat β catenin	Probe	5′-/56-FAM/ACGCCCTCC/ZEN/ACGAACTGC/3IABkFQ/-3′	9-10	NM_053357
	Primer2	5′-ACCCTTCAACTATCTCCTCCA-3′		
	Primer1	5′-GTACGAGCACATCAGGACAC-3′		
rat N cadherin	Probe	5′-/56-FAM/TCCCGGCGT/ZEN/TTCATCCATACCAC/3IABkFQ/-3′	14-15	NM_031333
	Primer2	5′-CCTCTCCTCCACCTTCTTCA-3′		
	Primer1	5′-GCTGATCCTTGTCCTCATGT-3′		
rat STAT3	Probe	5′-/56-FAM/TCGACCTAG/ZEN/AGACCCACTCCTTGC/3IABkFQ/-3′	14-16	NM_012747
	Primer2	5′-CTGACAGATGTTGGAGATCACC-3′		
	Primer1	5′-TGTGATGCCTCCTTGATTGTC-3′		
rat HIF1A	Probe	5′-/ATACCAGCA/ZEN/GTAACCAGCCGCA/3IABkFQ/-3	5-6	NM_024359
	Primer2	5′-GTTCACAAATCAGCACCAAGC-3′		
	Primer1	5′-GAACATCAAGTCAGCAACGTG-3′		

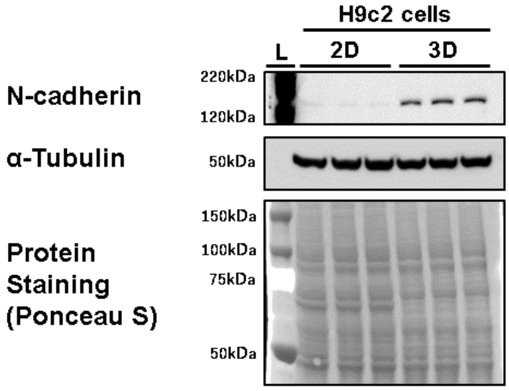

Supplemental Figure S1. Western blot analysis of N-cadherin of 2D and 3D cultured H9c2 cells.

Cell lysate of non-treated (NT) 2D or 3D H9c2 containing approximately 15 μg proteins was subjected to each lane of 4-12% gradient polyacrylamide gel and thereafter blotted onto a PVDF membrane (Millipore, Bedford, MA). After blocking with 5 % nonfat dry milk in TBS-T buffer, the membrane was sequentially incubated with 1st antibody (1:1000 dilution) of anti-N-cadherin rabbit polyclonal antibody (#4061, Cell Signaling Technology, Danvers, MA, U.S.A.) or anti-α-tubulin rabbit polyclonal antibody (#4074, Abcam, Cambridge, England) and 2nd antibody (1:5000 dilution) of anti-rabbit IgG/HRP-linked Antibody (#7074, Cell Signaling Technology, Danvers, MA, U.S.A.). Immunolabeling proteins were visualized by an Immobilon Western detection kit (Millipore, Billerica, MA). Representative immunolabeling and protein staining by Ponceau S of the transfer membrane are shown (n=3). L: ladder protein makers.
